# Ruling out nosocomial transmission of *Cryptosporidium* in a renal transplantation unit: case report

**DOI:** 10.1186/s12879-016-1661-5

**Published:** 2016-08-02

**Authors:** J. Brunet, J. P. Lemoine, B. Pesson, S. Valot, M. Sautour, F. Dalle, C. Muller, C. Borni-Duval, S. Caillard, B. Moulin, A. W. Pfaff, R. Razakandrainibe, A. Abou-Bacar, L. Favennec, E. Candolfi

**Affiliations:** 1 Laboratoire de Parasitologie et de Mycologie Médicale, Plateau Technique de Microbiologie, Hôpitaux Universitaires de Strasbourg, 1 place de l’Hôpital, BP 426, F-67091 Strasbourg cedex, France; 2 Institut de Parasitologie et Pathologie Tropicale, EA 7292, Fédération de Médecine Translationnelle, Université de Strasbourg, 3 rue Koeberlé, F-67000 Strasbourg, France; 3 Laboratoire de Parasitologie et de Mycologie, Plateau Technique de Biologie du CHU Dijon, 2 rue Angélique Ducoudray, BP 37013, F-21070 Dijon cedex, France; 4 UMR 1347, Université de Bourgogne, 17 rue de Sully, F-21000 Dijon, France; 5 Département de Néphrologie et Transplantation, Hôpitaux Universitaires de Strasbourg, 1 place de l’Hôpital, BP 426, F-67091 Strasbourg cedex, France; 6 Laboratoire de Parasitologie-Mycologie, EA 3800, Centre Hospitalier Universitaire, Université de Rouen, 1, rue de Germont, F-76031 Rouen, France

**Keywords:** *Cryptosporidium*, Renal transplant, Zoonotic species, Genotypic species identification, Case report

## Abstract

**Background:**

*Cryptosporidium* spp. is a ubiquitous parasite affecting humans as well as domestic and wild vertebrates, causing diarrhea in both immunocompetent and immunocompromised hosts worldwide. Its transmission occurs primarily by the fecal-oral route. In humans, *C. parvum* and *C. hominis* are the most prevalent species, whereas immunocompetent and immunocompromised individuals can also be infected by other zoonotic species*.* Renal transplant patients are prone to develop cryptosporidiosis, which can induce severe and life-threatening diarrhea.

**Case presentation:**

We report here a series of nearly concomitant cases of acute symptomatic cryptosporidiosis in three renal transplant patients attending the Strasbourg University Hospital Nephrology Unit. The clinical presentation was persistent diarrhea and acute renal failure. The diagnosis was confirmed by microscopic stool examination using a modified Ziehl-Neelsen staining method and species identification by molecular tools. All patients were treated with nitazoxanide and recovered from diarrhea after 14 days of therapy.

**Conclusion:**

Genotypic species identification was not consistent with an epidemic context, thus underlining the need for genotyping to monitor at risk patients.

## Background

The coccidian protozoan *Cryptosporidium* spp. is an intestinal parasite and a significant cause of enteric disease in humans and numerous other vertebrates worldwide. Cryptosporidiosis is the most common zoonotic cause of human parasitic diarrhea (*i.e.,* 60 % of epidemic cases linked to waterborne and 2–6 % of cases involving severe diarrhea worldwide), especially in immunocompromised individuals and young children [1]. The latter is notably the case in developing countries, where this parasite ranks second in the causes of death in children under 2 years [1, 2]. The prevalence of *Cryptosporidium* in stools of immucompetent persons was found to be lower in high-income countries than in developing regions [3]. Infection occurs by oocyst-stage ingestion *via* contaminated drinking water, food or recreational waters, as well as by direct or indirect human-to-human or animal-to-human contact [4]. In France, 407 cases of cryptosporidiosis were diagnosed between 2006 and 2009 [5]. A study conducted by the Strasbourg University Hospital between 2011 and 2013 detected *Cryptosporidium* spp. in 2.4 % of stools in which parasites have been detected out of a total of 6515 analyzed stools [6]. Over the past 20 years, three cryptosporidiosis outbreaks have been reported in France [5]. Other documented cases are linked to outdoor activity, swimming pools, day-care centers, and travel [3, 7]. Cryptosporidiosis can spread also among hospitalized patients and hospital staff and nosocomial outbreak of *Cryptosporidium* have been described. Source of infection could be contaminated water or contact with the hands of infected people [8, 9].

The increasing frequency of human cryptosporidiosis outbreaks raises relevant public health and economic concerns [10–12]. Human cases are commonly due to two species: *C. hominis*, which primarily infects humans, and *C. parvum*, which infects both humans and animals. Occasional infections by other species/genotypes, such as *C. felis*, *C. meleagridis*, *C. canis* or chipmunk and rabbit genotypes have been primarily reported in immunodeficient patients [5]. *C. hominis* and the zoonotic *Cryptosporidium* species are associated with a variety of clinical manifestations in humans [13].

The severity and duration of human *Cryptosporidium* infections are linked to the host’s immune status [14]. Immunocompetent patients experience self-limiting disease, while in immunosuppressed patients, especially those with T-cell deficiency, cryptosporidiosis is often chronic and severe with risks of extra-intestinal disease development [15].

In renal transplant patients, post-transplant cryptosporidiosis with diarrhea is a frequent complication [16]. In France, 69.3 % of clinically apparent cryptosporidiosis cases reported from 2006 to 2009 involved immunocompromised patients and 16.5 % of them were reported in patients who had received solid-organ or stem-cell transplants [5]. One report from a pediatric renal transplant unit demonstrated that infections were the primary cause of diarrhea, with *Cryptosporidium* spp*.* diagnosed in 11 % of 199 cases [3].

We report here three *Cryptosporidium* spp. infections with acute diarrhea and abdominal pain, observed almost simultaneously in three renal transplant patients who were subject to species genotyping in order to investigate a potential epidemic context in an outpatient nephrology unit.

## Case presentation

The three cases were diagnosed in the outpatient unit of the Nephrology Department at Strasbourg University Hospital, France.

### Clinical histories

Patient #1 was a 60-year-old man who underwent transplantation at the age of 52 for chronic renal failure due to polycystic kidney disease. He initially received immunosuppressive treatment, consisting of tacrolimus (4 mg/day), mycophenolate mofetil (MMF) (1 g x 2/day), and prednisone (7.5 mg/day). Eight years after renal transplantation, he presented with watery diarrhea, nausea and vomiting starting 15 days before consulting (September 25th, 2014). Physical examination revealed asthenia, weight loss (6Kg), hypotension, dry mouth, and acute renal failure (glomerular filtration rate [GFR]: 30 ml/min/1.73 m^2^). The patient reported no recent travel or contact with swimming pool water, non-drinking water or farm animals, but admitted to own a dog. No other family member experienced diarrhea.

Patient #2 was a 64-year-old man of Malian origin who had lived in France for 40 years and undergone transplantation at the age of 62 for chronic renal failure secondary to glomerulonephritis. Immunosuppressive treatment consisted of tacrolimus (7 mg x 2/day), MMF (750 mg x 2/day), and prednisone (10 mg/day). Two years and 4 months following renal transplantation, he presented with watery diarrhea and abdominal pain lasting for 15–20 days (starting September 26th, 2014). Physical examination revealed weight loss (13Kg), esophageal pain, and acute renal failure (GFR: 36 mL/min/1.73 m^2^). The patient also presented leucopenia and neutropenia, initially attributed to an overdose of tacrolimus. He reported no previous contact with non-drinking water, swimming pool water or farm animals, but had travelled to Mali for 2 months shortly before the onset of diarrhea. No other person of his family experienced diarrhea.

Patient #3 was a 34-year-old man of Kosovar origin who underwent transplantation aged 24-year-old for an undetermined reason. Acute transplant rejection 2 years later led to a second transplantation in September, 2014. Immunosuppressive treatment consisted of tacrolimus (6 mg x 2/day), MMF (750 mg x 2/day), and prednisone (25 mg/day). Ten days following the second renal transplantation (September 21th, 2014), the patient exhibited watery diarrhea and abdominal pain. Physical examination indicated weight loss (10Kg) and acute renal failure (GFR: 16 ml/min/1.73 m^2^). The patient, who reported no contact with non-drinking water, swimming pool water or farm animals, had travelled to Kosovo for 1 month before transplantation. His 2-year-old daughter also presented with diarrhea from an unknown cause that lasted for 3 days. No stool analyses were done for the daughter.

### Parasitological investigations

For all three patients, stool examinations performed at the first consultation revealed the presence of *Cryptosporidium* oocysts, using a modified Ziehl-Neelsen staining method (5–10 oocysts/slide, >100 oocysts/slide, and 1–5 oocysts/slide for cases #1, #2, and #3, respectively). All stool samples were negative for the bacteria *Clostridium difficile*, *Salmonella*, *Shigella* but also for rotavirus and norovirus, and for parasites *Giardia* and microsporidia.

DNA was extracted from the stool samples using a NucliSENS easyMAG device (bioMérieux, Marcy l’Etoile, France) [17]. Briefly, it consisted of adding 400 mg of stool samples to 1 mL of NucliSENS lysis buffer in a tube containing ceramic beads (lysing matrix D; MP Biomedicals, Illkirch, France), disrupted in a FastPrep-24 grinder (MP Biomedicals) at maximum power for 1 min. After 10 min of incubation at room temperature to ensure complete lysis, the tubes were centrifuged at 10,000 × *g* for 10 min and extraction was performed with 250 μL of supernatant. Elution was performed at 70 °C with 100 μL of elution buffer. An in-house real-time polymerase chain reaction (PCR) assay was set up to enable the detection and identification of the most common *Cryptosporidium* species/genotypes [18]. To this end, we used a single reaction tube with fluorescence-labelled probes for the real-time detection of *Cryptosporidium,* in addition to melting curve analyses of PCR products to differentiate between the *Cryptosporidium* species/genotypes. We conducted the amplification of a 258 bp DNA fragment located in the 18S ribosomal ribonucleic acid (rRNA) gene (GenBank accession n°L16996; positions 80 to 337) using the following primers: Cry80F: 5’-GTTAAACTGCRAATGGCT-3’; Cry337R: 5’-CGTCATTGCCACGGTA-3’. The CryAnch-labelled hybridization probe (5’-CCGTCTAAAGCTGATAGGTCAGAAACTTGAATG-fluorescein-3’) hybridizes in a region that is conserved among all *Cryptosporidium* species and the CrySens labelled hybridization probe (5’-LCRed640-GTCACATTAATTGTGATCCGTAAAG-3’) hybridizes in a polymorphic region (nucleotides 260 to 264) with various mismatches. Thermocycling and fluorescence detection were performed by means of a LightCycler 2.0 system (Roche Diagnostics) in a final volume of 20 μL, using a Roche LC Faststart DNA Master HYPROBE (Roche Diagnostics) with 0.5 μM of each primer, 0.2 μM of hybridization probe, 0.5U of UNG (Roche Diagnostics), and 5 μL of extracted DNA. After applying 95 °C for 10 min, amplification was commenced consisting of 50 cycles of 10-sec denaturation at 95 °C, 15 sec of annealing at 50 °C (with a touchdown protocol beginning at 60 °C), and 15 sec of elongation at 72 °C. The fluorescent signal (640 nm) was detected following the annealing step of each cycle. Species/genotypes differentiation was based on differences in the melting temperatures of the PCR-probe complexes, which were determined based on the extent of complementation of the probes to the target strand of the PCR product. For the melting curve analysis, a quick denaturation step was performed at 95 °C followed by a 30-sec annealing step from 45 °C to 80 °C (ramp-up rate: 0.1 °C/s), with continuous detection throughout the ramp up. The technique was validated using *Cryptosporidium* DNA of a stool collection already characterized at the genus level and positive controls for *C. parvum* and *C. hominis* [12].

The five predominant human pathogenic *Cryptosporidium* species were identified based on their melting curve profiles (61.9 °C, 53.8 °C, 48.8 °C, 56.7 °C, and 51.8 °C for *C. parvum*, *C. hominis*, *C. felis*, *C. meleagridis*, and *C. canis*, respectively). Given that *C. cuniculus*, an emergent human species, possesses the same melting curve profile as that of *C. hominis*, with both species exhibiting the same DNA sequence at the hybridization probe locus, all isolates identified as *C. hominis* were then sequenced to differentiate *C. hominis* from *C. cuniculus*.

For sub-genotyping analysis, DNA samples were subjected to amplification of an 850-bp fragment of the *gp60* gene using a nested PCR method [19]. The total volume of PCR mixture was 50 μL, containing 5 μL of DNA for the primary PCR or 5 μL of the primary PCR products, primers (outer primers: AL3531 and AL3535; inner primers: AL3432 and AL3534) at a concentration of 0.4 μM, 0.2 mM deoxyribonucleotide triphosphate mix, and 1.25U of DreamTaq DNA polymerase. Each PCR reaction was subjected to 40 cycles of 30s denaturation at 95 °C, 60s annealing at 55 °C, and 60s extension at 72 °C, with an initial 5 min denaturation at 95 °C and a final 10 min extension at 72 °C. PCR products were visualized by electrophoresis on an ethidium bromide stained 2 % agarose gel electrophoresis. Amplicons were purified and sequenced in both directions with the forward and reverse primers used in the secondary PCR at a concentration of 0.32 μM. Sequencing was performed using a BigDye Terminator v3.1 Cycle Sequencing Kit (Applied Biosystems, USA) and an ABI PRISM 3100 Genetic Analyzer® (Applied Biosystems, USA). We analyzed the quality of the generated electrophoregrams from each strand using 4Peaks software and compared them with those available in the GenBank database using the basic local alignment search tool. Subtype assignment was based on the number of trinucleotide repeats (TCA, TCG or TCT) in the coding for serine [20].

### Results

For Patient #1, genotyping revealed a *C. felis* infection*.* For Patient #2, genotyping revealed *C. hominis* sub-genotype IaA13*.* For Patient #3, the *C. parvum* sub-genotype IIaA13G1R2 was identified.

### Treatment

All three patients were treated with nitazoxanide (500 mg x 2/day for 14 days). For Patient #1, the stools tested negative 2 weeks after treatment initiation, with no recurrence of diarrhea observed 4 months after the first episode. For Patient #2, a reduction of tacrolimus was initiated and the diarrhea regressed 8 days after treatment initiation, although 3 months after therapy was started, his stools still tested positive. A second administration of nitazoxanide was thus prescribed and we requested a stool sample from his daughter for testing. One month after the second treatment course, his stools were tested negative for *Cryptosporidium* oocysts. For Patient #3, tacrolimus was also reduced and his diarrhea was regressing 1 month after treatment initiation. Four months after the second treatment course, his stools were tested negative for *Cryptosporidium* oocysts.

## Conclusions

We report here a series of nearly concomitant cases of acute symptomatic cryptosporidiosis in three renal transplant patients attending the same outpatient unit of a Nephrology Department. The patients’ consultations records in the Nephrology department showed that they could have been in contact in July 2014. This possibility of contact before the onset of symptoms suggesting a possible nosocomial infection requires a genotyping to explore this hypothesis. In these patients, *Cryptosporidium* species and *gp60* genotypes, which were determined to document a possible outbreak, provided no evidence of nosocomial transmission.

As our report demonstrates, the detection of three different *Cryptosporidium* species in three cryptosporidiosis patients excluded the possibility of nosocomial transmission in the Nephrology unit, where renal transplant patients frequently consult and come into contact with each other*.* Our findings highlight the risk of symptomatic cryptosporidiosis in immunosuppressed renal transplant patients. In 2014, nine out of ten patients with cryptosporidiosis diagnosed by the medical Parasitology and Mycology laboratory of the Strasbourg University Hospital were renal transplant patients (unpublished data). In a pediatric renal transplantation unit, *Cryptosporidium* spp. was confirmed as the principal cause of diarrhea in patients between 6 months and 12 years of age following transplantation. In Poland and India, the prevalence of *Cryptosporidium* spp. in renal transplant patients was reported to be 18.8 and 20 %, respectively [21, 22]. *Cryptosporidium* spp. infections were more commonly associated with profuse watery diarrhea in solid-organ recipients than in immunocompetent patients [21, 23, 24]. Our patients undergoing combined immunosuppressive therapies exhibited watery diarrhea for several weeks before consulting, suggesting that the prevalence of *Cryptosporidium* spp. infections is probably underestimated in renal transplant units where screening of patients with diarrhea is not routinely performed. In all three of our patients, the symptoms completely resolved within 8 days to 1 month, in line with previous reports of slower recovery duration compared to immunocompetent patients in whom diarrhea symptoms usually cease after 10 to 15 days without treatment [3, 16]. Considering the role of immunosuppression in the appearance and persistence of cryptosporidiosis, we opted to reduce the immunosuppressive regimen in two of our patients, which, in association with the anticryptosporidial agent, could prove an effective method in reducing both duration and severity of symptoms [3, 12, 25].

*C. felis* cryptosporidiosis (patient #1) is rarely diagnosed in France (4.8 % of all cases between 2006 and 2009 *vs.*, 54 % for *C. parvum* and 36 % for *C. hominis*) [5]. No contact with cats was reported, in agreement with previous reports showing that cat ownership is not a significant risk factor for *C. felis* infection and that *C. felis* host specificity is not very strict, since it was observed in cats, cattle and humans, thus rendering it often difficult to determine the source of infection [5, 26, 27].

*C. hominis* infection observed in Patient #2, is prevalent worldwide, and especially in developing areas, with similar incidences to those of *C. parvum* infection in most European countries, but less frequently reported than *C. parvum* infection in France and the Middle-East area [20, 24–29]. Travel-related cryptosporidiosis and small family outbreaks have been frequently associated with *C. hominis* infection, consistent with the onset of symptoms during our patient’s trip to Africa [5]. To the best of our knowledge, human cases of the *C. hominis* IaA13 sub-genotype have only been reported in Australia, but reports of the *C. hominis* genotypes present in Africa are scarce [30].

*C. parvum*, the predominant species in French cryptosporidiosis patients, was detected in Patient #3 [5]. The *C. parvum* IIaA13G1R2 genotype had previously been reported in Sweden and Germany with no identification of the mode of contamination [31].

Our findings confirm the need to consider cryptosporidiosis as a significant cause of acute persistent watery diarrhea in immunocompromised kidney transplanted patients. In hospitals or day-care centers, renal transplant patients should be informed in order to minimize risk of infection by handwashing and avoiding contact with young pets, infected people and swimming pools. Moreover, in renal transplant unit, patient with prolonged diarrhea should be tested for *Cryptosporidium* and isolated [14, 15]. Risk of diarrhea due to *Cryptosporidium* being high for kidney transplant patients, species identification by molecular biology is important. First, to eliminate a possible nosocomial infection in patients who will be often in contact during consultations in the nephrology department; second, to determine the source of contamination (notably animal) and eliminate it to prevent any subsequent recontamination.

## Abbreviations

MMF, mycophenolate mofetil; GFR, glomerular filtration rate; PCR, polymerase chain reaction; rRNA, ribosomal ribonucleic acid
